# Impact of Sensitization on School Teachers' Knowledge and Attitudes Towards Adverse Drug Reaction Reporting During National Deworming Day: A Prospective Quasi-Experimental Study

**DOI:** 10.7759/cureus.73209

**Published:** 2024-11-07

**Authors:** Babita Sheoran, Tirthankar Deb, Mayur Tuteja, Ashish Kumar, Alapan Das, Nitika Sharma

**Affiliations:** 1 Pharmacology, Kalpana Chawla Government Medical College, Karnal, IND; 2 Pharmacology, All India Institute of Medical Sciences, Kalyani, Kalyani, IND; 3 Community Medicine, Kalpana Chawla Government Medical College, Karnal, IND

**Keywords:** adverse drug reaction, albendazole, attitude, knowledge, mass administration, national deworming day, pharmacovigilance, sensitization

## Abstract

Background: School teachers play a crucial role in executing administration of single dose albendazole to school children on National Deworming Day (NDD). Effective reporting of adverse drug reactions (ADRs) during such mass administration of medicines at the time of such public health programmes is vital for several policy decisions on safety. The study aims to evaluate teachers' knowledge and attitudes on ADR reporting regarding NDD and assess the impact of a sensitization program.

Method: A total of 163 school teachers from 94 schools in the district, who gave informed consent were included. The study utilized a pretested and prevalidated questionnaire, scored on a 0-10 scoring scale. Employing a prospective quasi-experimental one-group pre-test and post-test research design, the primary aim was to evaluate the impact of the awareness program, with secondary objectives focused on demographic correlations with program effectiveness.

Results: The study shows a significant improvement in participants' scores from the pre-test (mean score of 5.60) to the post-test (mean score of 8.28), resulting in a mean deviation of -2.68±1.74 (p < .001), suggesting that the intervention significantly enhanced outcomes. Additionally, the results indicate that the mean post-test score was slightly higher among urban participants in comparison to rural participants, with mean scores of 8.45 and 8.10, respectively.

Conclusion: The sensitization program significantly improved the knowledge and attitude of school teachers engaged in NDD regarding ADR reporting. However, long-term studies on the impact of this improvement in knowledge and attitude on actual ADR reporting need to be planned.

## Introduction

Soil-transmitted helminths (STHs), including hookworm (*Necator americanus* and *Ancylostoma duodenale*), whipworm (*Trichuris trichiura*), and roundworm (*Ascaris lumbricoides*), are parasitic worms that substantially torment people in tropical areas, especially in surroundings with scarce resources [1]. School-age children in developing countries are particularly vulnerable to these infections due to frequent barefoot activities, limited access to clean water, and poor hygiene that increases the threat of contact with polluted soil. STHs affect the development, aliment, and general well-being of children, adversely. World Health Organization (WHO) championed a pivotal strategy that was school-based preventative chemotherapy with Albendazole, a broad-spectrum anthelmintic medication, as an affordable and practical strategy in the international endeavor to manage and eradicate STH, a grave public health problem. Mass drug administration (MDA) programs are generally employed to administer albendazole [2-4]. Ministry of Health and Family Welfare (MoHFW) established the National Deworming Day (NDD) in February 2015 as a vital element of the National Health Mission. Treating implicit parasitic worm infections in kids between the ages of one to nineteen is the primary objective of NDD. This comprehensive initiative encompasses government schools, government-aided schools, private schools, as well as Anganwadi centers [5]. Adverse event (AE) surveillance is a reliable way of monitoring the safe functioning of preventative chemotherapy programs and MDA programs and enhances their legitimacy [6]. The Pharmacovigilance Programme of India (PvPI) received adverse drug reaction (ADR) reports at a significantly advanced rate since systematic training and awareness programs along with the introduction of the "PvPI Drug Safety Newsletter" [7-9]. The pharmacovigilance indicators of WHO are essential for managing public health enterprises successfully. These indicators cover a wide range of ways to monitor the efficacy and safety of medications used in healthcare systems. These include standardizing ADR reporting forms to cover various aspects of medication use, monitoring the total number of ADR reports, therapeutic ineffectiveness reports, hospital admissions, and drug-related deaths per 1000 individuals annually, and integrating pharmacovigilance activities into functional documents and treatment guidelines. The indicators also stress how crucial it is to send in completed reports to the WHO database and public pharmacovigilance centers so that they may be thoroughly examined and taken further. Public health enterprises can ameliorate patient issues, detect new problems, and achieve pharmaceutical safety by following these parameters. Pharmacovigilance indicators are metrics used to evaluate the inputs, processes, outputs, outcomes, and impacts of health system development initiatives, programs, or services related to drug safety monitoring. They provide evidence to estimate the degree to which a pharmacovigilance program is meeting its objectives. Pharmacovigilance is essential to public health activities because it guarantees the prudent and safe use of pharmaceuticals, the fast identification of any unfavorable drug interactions, and the implementation of prompt measures to lessen the impact of any potential adverse event [10-13].

In public health programs, ADR underreporting is quite common, resulting in an inaccurate depiction of their prevalence. This can be attributed to the absence of clear pharmacovigilance mandates, inadequate tools, and inefficient reporting systems. Without proper structures in place, healthcare providers may not prioritize ADR reporting, hindering surveillance efforts and patient safety. Addressing these gaps is crucial to improving ADR monitoring and ensuring the safe use of medications in public health programs [13,14]. The implementation of NDD and ADR reporting in the schools following albendazole administration is greatly aided by school teachers [4,5]. Thus, they must retain an acceptable understanding of the program and ADR reporting. This can only get better if the medical fraternity periodically organizes sensitization campaigns. The cooperation between educators and the medical community via sensitization initiatives becomes a vital element in raising public health knowledge and improving the educational system's response to public health priorities [15-17]. Previous research has indicated a link between healthcare workers' underreporting of ADRs and knowledge and attitude deficiencies [18-22]. Considering the significant part that school teachers play in the implementation of NDD, it's critical to recognize and address any knowledge gaps that they may have concerning ADR reporting. The study aims to evaluate the knowledge and attitudes of school teachers engaged in albendazole administration in the NDD program towards adverse drug reaction reporting and to assess the impact of a comprehensive sensitization program regarding the same. The study intends to identify potential challenges, gaps, and opportunities for improving pharmacovigilance practices within the context of NDD. By bringing these variables to light, we hope to ameliorate the effectiveness of teacher-led interventions and support systems that enable them to identify, record, and communicate adverse responses in a way that eventually protects the health and safety of students taking the medicine.

## Materials and methods

The study was conducted after approval from the Institutional Ethics Committee, Kalpana Chawla Government Medical College, Karnal (Approval No. KCGMC/IEC/2023/120). The study adhered to ethical standards by maintaining participant confidentiality throughout the research process. The study duration was three months (January to March 2023). This study was done in various government schools involved in NDD in the Karnal district of Haryana, India. Visits and sensitization programs in the schools were conducted from the first week of January 2023 to the first week of February 2023, thus completing the process before the NDD which was scheduled on February 10, 2023, with a mop-up round on February 17, 2023. A comprehensive list of government schools provided by the Department of District Education, Karnal, Haryana was obtained. Under the NDD program, each cluster of schools covering around four to six schools is overseen by a headmaster of one of these schools who is designated as the cluster head as part of the administrative policy of the government. Out of 96 already available clusters of schools in the district, 20 such clusters were randomly selected for the study following convenient sampling. These 20 clusters included a total of 94 schools. Covering these 94 schools, a total of 163 participants were involved in the study including cluster heads and other school teachers (directly engaged in NDD program) who expressed willingness to participate in the study and provided written informed consent.

Following the principles of training as embedded in the six-step approach "TTWVAA" (team formation, train the trainers, work distribution, visits, active extraction, and assisted reporting) of Deb’s active surveillance and assisted reporting system of ADR monitoring, a team of trainers was formed among the investigators and prior training was provided to them on conducting the sensitization program for school teachers [23]. Working on the same innovative approach, work distribution was done among this team. One of the investigators was assigned to build communication with the education department to collect data on cluster heads of schools in the district. Another investigator telephonically communicated with the heads of the clusters, and as per their willingness and convenience of both sides, a schedule for trainer visits and sensitization of school teachers was prepared. Following this pattern, visits and training sessions were scheduled at 20 locations, i.e., school premises leading these 20 clusters. Another investigator coordinated with the medical college administration to arrange transport facilities for the visits. A predesigned and prevalidated sensitization program was conducted on every visit. It was attended by teachers from each school within the particular cluster who were entrusted with albendazole administration in that school under NDD. The sensitization program of around 90 minutes was structured around four sections, each being covered in around 15 minutes for the first two sections and 30 minutes each for the other two sections, namely (a) basic understanding of ADR, (b) significance of reporting ADRs, especially in context of NDD, (c) process of reporting adverse events using ADR reporting form of Pharmacovigilance Programme of India (PvPI), and (d) brief understanding of albendazole and its possible adverse drug reactions. A prepared PowerPoint presentation along with an interactive mode of discussion was used. The team carried a portable projector for the same. Informed consent was obtained using an informed consent form.

The study employed a pre-validated and pre-tested questionnaire with 10 questions, formulated in both English and Hindi languages (see Appendices). Responses were recorded as scores based on a zero to 10 scale. A quasi-experimental design involving one-group pre-test and post-test research was adopted for this study. This methodological framework aimed to provide a comprehensive analysis of the effectiveness of the sensitization program. Pre- and post-test responses were obtained via Google Forms to overcome the observer bias. The outcome of the study was to analyze the impact of the sensitization initiative.

## Results

The sociodemographic details of the participants are shown in Table 1. The majority, constituting 50.3% (N=82), out of a total of 163 participants, were in the 41 to 50 years age group. 31 to 40 years age group comprised 23.9% (N=39) of the studied participants, while 24.5% (N=40) fell within the 41 to 50 years range. Notably, the 20 to 30 years age group had the lowest representation, accounting for only 1.2% (N=Two) of the overall participants. In terms of gender distribution, the study had a nearly equal division among the participants, with 47.9% (N=78) identified as male and 52.1% (N=85) as female. Most participants in the study held post-graduate qualifications, accounting for 65.6% (N=107). Additionally, 24.5% (N=40) had undergraduate degrees, while only 9.8% (N=16) were qualified beyond the post-graduate level. The study participants had a nearly equal distribution between rural residents, constituting 47.9% (N=78), and urban residents, comprising 52.1% (N=85) of the sample (Table 1).

**Table 1:** Sociodemographic details of study participants (N=163) The data has been represented as N (%)

Factors	Frequency (N (%))
Age group (years)	
20-30	2 (1.2)
31-40	39 (23.9)
41-50	82 (50.3)
51-60	40 (24.5)
Gender	
Male	78 (47.9)
Female	85 (52.1)
Educational qualification	
Graduation	40 (24.5)
Post-graduation	107 (65.6)
Higher post-graduation	16 (9.8)
Residence type	
Rural	78 (47.9)
Urban	85 (52.1)

**Figure 1:**
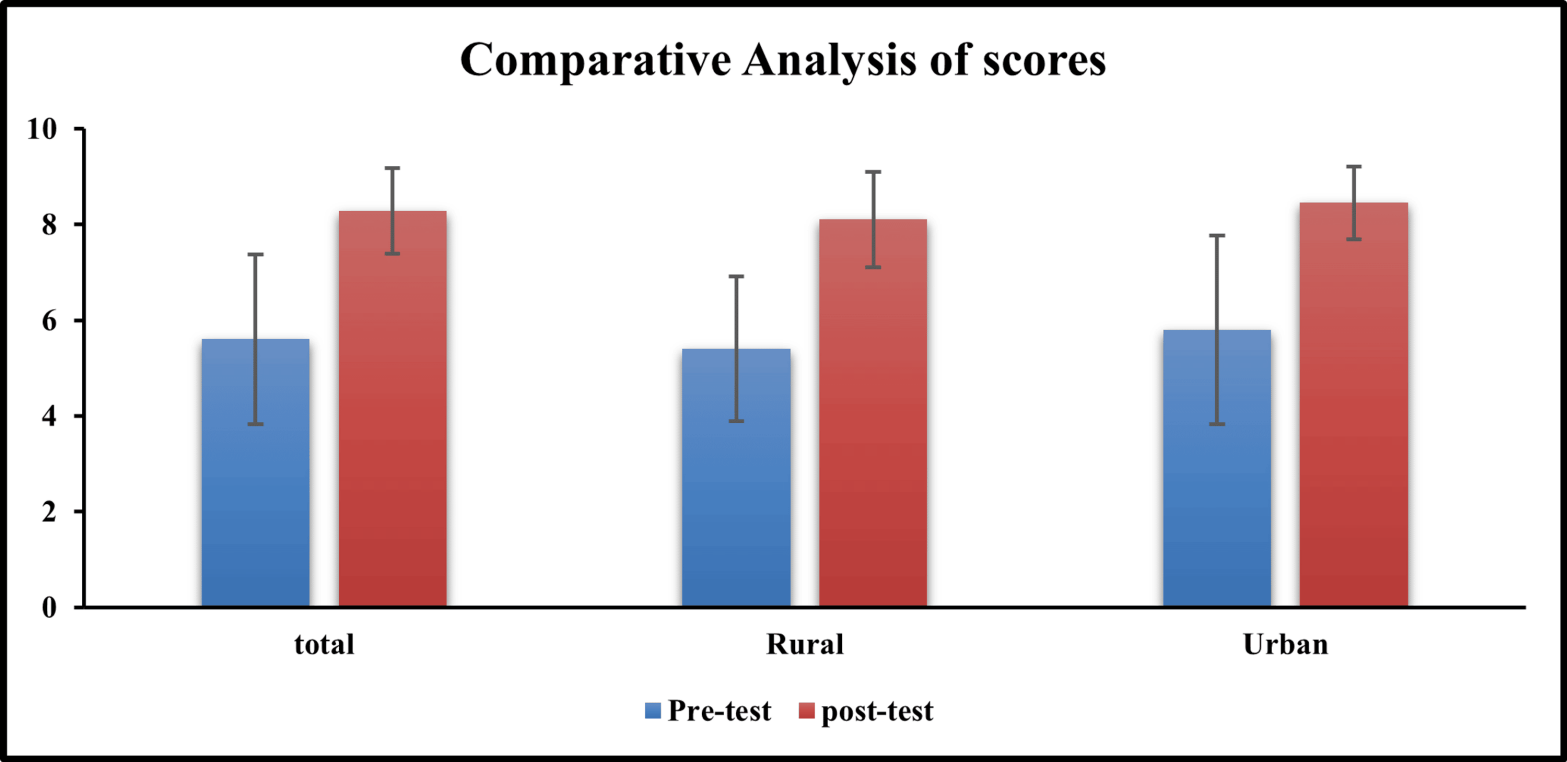
Comparative analysis of pre and post-test scores among participant (overall, rural and urban area) The data has been represented here as Mean±SD, (p<0.001)

The study findings indicate that the mean pre-test score of the study subjects was 5.60±1.77. In contrast, the mean post-test score was 8.28± 0.90 (Figure 1). Notably, the mean difference between the pre-test and post-test scores is -2.68±1.74 which is significant statistically (p < .001) (Table 2). The analysis of the sensitization program among studied participants shows significant improvement in both rural and urban regions as evidenced by the pre and post-test scores. In rural areas, the mean pre-test score was 5.40 while the mean post-test score was 8.10. Whereas, in urban areas, the mean pre-test score was 5.79 with a mean post-test score of 8.45. The results showed that the mean post-test score was higher in urban areas compared to rural areas (8.45 vs 8.10). However, this difference was statistically insignificant (p>.05).

**Table 2:** Comparison of the test scores Data has been represented here as Mean±SD, *p<0.001

Score of the participants	Overall (N=163)	Rural (N=78)	Urban (N=85)
Pretest	5.60±1.77	5.40±1.51	5.79±1.97
Posttest	8.28±0.90	8.10±1.00	8.45±0.76
Mean difference	-2.68±1.74*	-2.705±1.387*	-2.659±2.015*

## Discussion

Many studies have been done to assess the knowledge gap and awareness of doctors or other healthcare professionals like nurses or pharmacists regarding ADR reporting but no such study that includes school teachers has been done yet. This deficiency of data attains importance because school teachers are directly involved in the implementation of several public health programs in India. Hence, their knowledge and attitude are vital to ensure the safety of beneficiaries of such MDA programs. The present study results were closely related to a study published by Tabali et al. in 2009, which demonstrated that educational interventions can augment physician awareness of ADRs [16].

Similarly, a study by Deb et al. in 2019 indicated substantial improvement in ADR reporting, both qualitatively and quantitatively post-sensitization, with scores improving from 36 to 77, over three months among clinicians and nursing staff [17]. The findings suggest that regular education is a vital tool for enhancing perception and awareness about ADRs. Based on this it is recommended that annual repetition of such educational involvements has to be done [17]. A study done by Ahmad et al. showed that Indian pharmacists exhibit inadequate knowledge, attitude, and practice (KAP) toward reporting ADR and pharmacovigilance. However, with supplementary training in pharmacovigilance, pharmacists working across various sectors in India can be integrated into the ADR reporting system [24]. Several similar studies have demonstrated the significant impact of sensitization programs on the understanding, attitudes, and behaviors among parents and teachers regarding STH control programs for school children [25-27]. A study done by Njomo et al. demonstrated that preschool teachers can also help to raise awareness in the community regarding controlling STH [28].

Training programs have been shown to not only provide school teachers with basic knowledge on helminth infections but also empower them to knowledgeably execute drug administration exercises [29]. While these previous studies have shown the impact of sensitization on even teachers regarding the implementation or coverage of MDA or STH control programs, the present study goes a step ahead by evaluating the influence of such sensitization on their knowledge and attitude in relation to ADR reporting which defines the shield of the safety of beneficiaries in such public health programs. Till now, ADRs have predominantly been reported by healthcare workers. However, in national public health programs in India such as NDD, school teachers play a pivotal role as they directly administer Albendazole tablets or syrup to students under their supervision. Hence, they need to be well-informed not only about the correct administration of the medicines but also regarding detecting and using accurate methods and channels for timely reporting any ADRs experienced.

This study also has some limitations. Firstly, the study only analyses the immediate effect of sensitization, while long-standing effects, especially in terms of practices of the participants in relation to ADR reporting could not assessed. Further long-duration studies could be carried out to assess the impact on practices, along with exploring whether annual training sessions might yield better outcomes due to reinforcement. The small sample size and study design fail to permit for controlling external interventions at the individual level, like individual courses, internet-based information, etc. which could potentially influence our findings. However, this study draws attention to the aspect of safety in public health programs of MDA through a workforce of ground-level service providers like teachers well-trained in ADR reporting. A robust safety mechanism in place is expected to enhance public trust and participation in government-run health programs. The fact that this study incorporates sensitizing school teachers is one of its strengths because there are not many studies on the ADR reporting related sensitization for MDA programs like NDD, and even fewer on teachers or other community workers engaged in such mass drug administration under public health programs.

## Conclusions

The program of sensitisation enhanced the understanding and disposition of educators involved in NDD about ADR reporting, resulting in a noteworthy distinction in the results of the pre-and post-tests. This study can serve as a foundation for developing a comprehensive training programme on ADR reporting targeted at end-level service providers like school teachers and integrating the same into public health programmes. Long-term studies on the impact of such a sensitisation programme on actual ADR reporting and safety of beneficiaries are future scope of research in this field.

## Appendices

Questionnaire 

Section 1. Demographic Data

1.       Email:

2.       Full Name/ पूरा नाम:

3.       Age/ उम्र:

4.       Gender/ लिंग:

a.       Male/ पुरुष

b.       Female/ महिला

c.       Prefer not to say/ टिप्पणी नहीं करना पसंद करते हैं

5.       Contact number/ मोबाइल नंबर:

6.       Highest Educational Qualification/ उच्चतम शैक्षिक योग्यता:

7.       Place of posting/ पोस्टिंग की जगह:

8.       Designation/ पदनाम:

Section 2: General Question

1.       Are you aware about the National Deworming Day?

क्या आप राष्ट्रीय कृमि मुक्ति दिवस के बारे में जानते हैं?

a.       Yes/हाँ

b.       No/नहीं

2.       When is National De-Worming Day celebrated?/राष्ट्रीय कृमि मुक्ति दिवस कब मनाया जाता है?

a.       10th of Feb every year/हर साल 10 फरवरी

b.       as per guidelines from MoHFW/ MoHFW के दिशानिर्देशों के अनुसार

3.       Do you know that Albendazole is given to children under N.D.D.?

क्या आप जानते हैं कि एनडीडी के तहत बच्चों को एल्बेंडाजोल दी जाती है। ?

a.       Yes/हाँ

b.       No/नहीं

4.       Select known and possible adverse effects of Albendazole?

एल्बेंडाजोल के ज्ञात और संभावित प्रतिकूल प्रभावों का चयन करें

a.       Abdominal Pain/पेट में दर्द

b.       Vomiting/उल्टी करना

c.       Dizziness/चक्कर आना

d.       Diarrhea/दस्त

e.       All of the above/ऊपर के सभी

f.        None of the above/इनमे से कोई भी नहीं

5.       Who can report adverse events following Albendazole administration?

एल्बेंडाजोल लेने के बाद प्रतिकूल घटनाओं की सूचना कौन दे सकता है?

a.       Health care professional/स्वास्थ्य देखभाल पेशेवर

b.       Educational institution professionals/शैक्षिक संस्थान के पेशेवर

c.       Patient encountering adverse events/रोगी को प्रतिकूल घटनाओं का सामना करना पड़ता है

d.       All of the above/ऊपर के सभी

6.       Whom should you report in case you suspect an adverse event following the usage of Albendazole?

एल्बेंडाजोल के उपयोग के बाद प्रतिकूल घटना का संदेह होने पर आपको किसे रिपोर्ट करना चाहिए?

a.       Cluster in-charge/क्लस्टर प्रभारी

b.       National co-ordinating centre/राष्ट्रीय समन्वय केंद्र

c.       ADR monitoring centre/एडीआर निगरानी केंद्र

d.       Any of the above/ऊपर का कोई भी

7.       Which of the National Program among the following is responsible for reporting of adverse drug reaction in India?

निम्नलिखित में से कौन सा राष्ट्रीय कार्यक्रम भारत में प्रतिकूल दवा प्रतिक्रिया की रिपोर्टिंग के लिए जिम्मेदार है?

a.       Pharmacovigilance programme of India (PvPI)/ भारत का फार्माकोविजिलेंस प्रोग्राम (PvPI)

b.       Adverse event monitoring centre/ प्रतिकूल घटना निगरानी केंद्र

c.       National Deworming Day programme/ राष्ट्रीय कृमि मुक्ति दिवस कार्यक्रम

d.       None of the above/ इनमे से कोई भी नहीं

8.       Where should you report adverse events?

आपको प्रतिकूल घटनाओं की रिपोर्ट कहां करनी चाहिए?

a.       Civil Hospital/ सिविल अस्पताल

b.       Nearby adverse event monitoring centre/ निकट प्रतिकूल घटना निगरानी केंद्र

c.       Online App/ ऑनलाइन ऐप

d.       Both b and c/ बी और सी दोनों

9.       Have you ever got the chance of witnessing and reporting of an adverse drug reaction in the past?

क्या आपको कभी अतीत में प्रतिकूल दवा प्रतिक्रिया देखने और रिपोर्ट करने का मौका मिला है?

a.       Yes/हाँ

b.       No/नहीं

10.   Why ADR reporting is important for general population?

आम जनता के लिए एडीआर रिपोर्टिंग क्यों महत्वपूर्ण है?

a.       To treat the reaction/ प्रतिक्रिया का इलाज करने के लिए

b.       For awareness so that better drugs can be used/ जागरूकता के लिए ताकि बेहतर दवाओं का इस्तेमाल किया जा सके

c.       To help the government authorities for better treatment protocols/ बेहतर उपचार प्रोटोकॉल के लिए सरकारी अधिकारियों की मदद करना

d.       All of the above/ ऊपर के सभी
